# Preprocedural Pool Testing Strategy for Dentistry during the COVID-19 Pandemic

**DOI:** 10.1177/2380084421989693

**Published:** 2021-01-21

**Authors:** F. Umer, A. Arif

**Affiliations:** 1 Department of Surgery, Aga Khan University Hospital, Karachi, Pakistan

**Keywords:** coronavirus infections/diagnosis, specimen handling, economics, clinical laboratory techniques, pandemics, polymerase chain reaction

## Abstract

**Introduction:**

Aerosol-generating procedures (AGPs) put the dental health care professionals (DHCPs) at a greater risk for acquiring severe acute respiratory syndrome coronavirus 2 (SARS-CoV-2) infection. In late June 2020, the Centers for Disease Control and Prevention advised elective dental procedures provision to asymptomatic patients while mandating strict infection control protocol and suggested the use of preprocedural testing as an adjunct. A cost-effective method for mass preprocedural testing is pool testing, which has specificity and sensitivity similar to polymerase chain reaction. This article aims to assess the outcomes and utility of incorporating preprocedural testing protocol for SARS-CoV-2 in dental clinics before providing AGPs.

**Method:**

The patients who were recommended AGPs where rubber dam placement was not possible were advised to undergo preprocedural testing for SARS-CoV-2. Pool testing strategy was employed, and patients were asked to get tested 48 h before the day of the procedure.

**Results:**

Out of a total of 1,000 patients, who presented from June 2020 to late July 2020, 464 were recommended dental procedures. In 194 of 464, AGPs could not be performed under rubber dam isolation; therefore, the patients were advised to get a preprocedural pool test. In total, 111 patients deferred the procedure and testing. Out of 83 who got tested, 7 were positive for SARS-CoV-2, 5 of whom were tested in early June 2020 and 2 in late July 2020.

**Conclusion:**

Pool testing within its limitations can be a useful preprocedure test in asymptomatic low-risk patients for AGP in dentistry, especially when the disease prevalence is low or moderate (<10%). It has the potential of reducing testing costs significantly while conserving reagent and other resources. Preprocedure testing, however, also gives rise to certain ethical concerns that also need to be addressed.

**Knowledge Transfer Statement:**

The results of this study can be used by clinicians when deciding which preprocedure testing approach they wish to use when performing aerosol-generating procedures in asymptomatic patients with consideration of cost sensitivity and specificity values.

## Introduction

Severe acute respiratory syndrome coronavirus 2 (SARS-CoV-2) was declared a global pandemic in March 2020, and since then, it has claimed millions of lives and trillions of dollars. Many countries had to impose lockdown for approximately 7 wk to slow the virus spread, but this negatively affected the economy in all sectors, especially the health care sector (Emanuel et al. 2020).

The most commonly known SARS-CoV-2 transmission route is through inhalation of respiratory droplets or aerosols from infected individuals (Jamal et al. 2020). In the dental clinic, aerosols are generated during dental procedures as a result of water irrigation for cooling of the dental handpieces and ultrasonic devices (Epstein et al. 2020). Furthermore, high viral loads have been found in the saliva of both symptomatic and asymptomatic infected patients. Thus, the dual effect of aerosol generation along with saliva and the fact that dental work requires close proximity and prolonged contact time leaves dental health care providers (DHCPs) particularly vulnerable to contract coronavirus disease 2019 (COVID-19) infection (Jamal et al. 2020). This led to confinement of dental work to emergency management only to reduce the risk of cross-infection, consequently leading to grave monetary losses in the dental practices (Schwendicke et al. 2020).

Pakistan received positive confirmation of SARS-CoV-2 cases in February 2020, which eventually led to strict lockdown enforcement (Tariq et al. 2020). However, during late May 2020, many countries, including Pakistan, eased lockdown restrictions, allowing the return of workflow to normalcy. This facilitated elective treatment in dentistry per Centers for Disease Control and Prevention (CDC) guidelines, which further suggested that “facilities can consider implementing preadmission or preprocedure diagnostic testing for SARS-CoV-2” (https://www.cdc.gov/coronavirus/2019-ncov/hcp/dental-settings.html).

This preprocedural testing is important because an asymptomatic/presymptomatic patient poses the greatest risk of contagion, especially while providing aerosol-generating procedures (AGPs) (Lauer et al. 2020). An ideal preprocedural test should be cost-effective, highly sensitive, and easy to perform, and it should generate rapid results. For SARS-CoV-2 diagnosis, real-time reverse transcription polymerase chain reaction test (RT-PCR) is the gold-standard test that is performed using respiratory samples. However, RT-PCR testing is expensive, time-consuming, and requires specialized infrastructure (Wu et al. 2020). A single RT-PCR test may cost approximately $41 in Pakistan and the United States (Mahony et al. 2004). Other alternative methods for preprocedure testing are antigen and antibody tests, which, although rapid, may suffer from suboptimal sensitivity (36.4%) (Pan et al. 2020). Furthermore, antibodies may take up to 12 to 14 d to develop; hence, the efficacy of the antibody test is reduced in the acute phase of infection when the patient is asymptomatic/presymptomatic and therefore has limited use as a preprocedural test in asymptomatic patients (Zhao et al. 2020). In contrast, the latest literature on pool sampling for COVID-19 screening has shown promising results with sensitivity values of 91%, which is close to RT-PCR (Mutesa et al. 2020). Furthermore, the pool testing strategy offers the advantage of reduced operational costs in certain circumstances by up to 20-folds while preserving reagents as well as specialized human resources (Mutesa et al. 2020). However, pool testing inherits all the aforementioned disadvantages of being an RT-PCR–based test.

Due to the low costs it offers to the patients while maintaining high sensitivity, it was decided that all those patients requiring surgical procedures under general anesthesia and AGPs without a rubber dam in dentistry would be required to undergo preprocedure pool testing.

It seems unlikely that an infected symptomatic individual would seek elective dental care, and therefore, the biggest challenge our profession faces is the risk of getting infected by asymptomatic spreaders. With the COVID-19 cases perpetually increasing, it is imperative in order for our profession to thrive that we come up with neoteric solutions to counter this hazard. In this communication, we have highlighted the concept of the pool testing, which was strategically used as a preprocedural test for patients requiring elective AGPs.

## Method

The dental clinic at our tertiary care hospital started elective dental treatment on May 28, 2020. Non- AGPs were carried out if the patient was asymptomatic after initial screening, and AGPs were done under rubber dam isolation. This was allowed because it has been demonstrated that rubber dam isolation can decrease aerosol generation by up to 70% within a 3-foot radius (Jamal et al. 2020). However, preprocedural testing was mandatory in procedures such as crown cutting, endodontic surgery, surgical extractions, and other AGPs in which rubber dam isolation was not feasible (Umer and Motiwala 2020).

The patients were counseled about the usefulness of preprocedure testing and asked to get the test done 48 h before the procedure. After the test was done, patients were asked to self-isolate for 2 d. To ensure cost-effective and reliable preprocedural testing, we employed the “pool testing” strategy.

To get tested, the patient was given an option of an appointment at the centralized hospital testing site or, if they had privacy or any other concerns, they could opt for at-home testing service for an additional service fee. Pooling samples were compiled on a first come, first serve basis in a central biosafety level III laboratory. The pool size was internally validated according to US Food and Drug Administration (FDA) policy for the COVID-19 test (https://www.fda.gov/media/135659/download), and each pool consisted of 6 samples (6:1 ratio) collected as nasopharyngeal swabs (Hogan et al. 2020).

These samples were stored at 4°C before processing for virus isolation and nucleic acid detection purposes. Furthermore, each specimen went through a process of batch organization and was given dual identification and a barcode through an electronic lab tracking system. They were also manually registered in the logbook so that samples were not mixed.

The test was run on a Cobas SARS-CoV-2, based on fully automated sample preparation (nucleic acid extraction and purification), followed by PCR amplification and detection. Selective amplification of target nucleic acid from the samples was achieved by the use of target-specific forward and reverse primers for ORF1 a/b nonstructural region that is unique to SARS-CoV-2. In addition, a conserved region in the structural protein envelope E-gene was chosen for pan-Sarbecovirus detection. The pan-Sarbecovirus detection sets also detect the SARS-CoV-2 virus. Finally, the sample was stored for another 48 h after reporting before being discarded.

According to the Dorfman algorithm, if the pool tests are negative, then all individuals in that pool have been efficiently tested with a single test, allowing conservative usage of reagents and finances, with results posted in 24 h (Wacharapluesadee et al. 2020). However, in the case of positive pool tests, at least 1 person in the group is tested positive for the disease, and each sample from the pool needs an additional test using RT-PCR. This added a delay of 24 h (Wacharapluesadee et al. 2020), and therefore all the patients were scheduled for dental procedures after 48 h from the date of the test (Figure).

**Figure.**
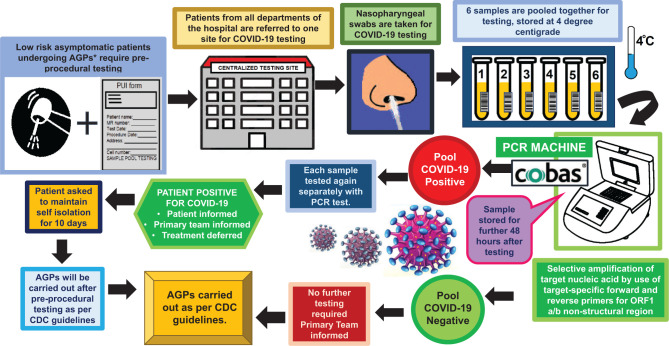
Workflow for preprocedure testing. *AGPs (aerosol-generating procedures) include crown cutting, surgical extraction, and scaling and polishing and have to be performed without a rubber dam.

## Results

From May 28, 2020, to July 20, 2020, a total of 1,000 patients had visited the dental clinics. Out of these, 536 patients received consultation only. Procedures were recommended for 464 patients, which included non-AGPs, AGPs under rubber dam isolation, and AGPs after preprocedural testing (Table). Out of 464 procedures, 270 patients had undergone treatment without the need for preprocedural testing (AGPs under rubber dam isolation and non-AGPs), whereas 194 (out of 464) patients were advised AGPs in which rubber dam placement was not feasible, and hence preprocedural testing was recommended. Out of these 194 patients, 111 patients chose not to get preprocedural testing, and the remaining 83 got testing done, out of whom 76 patients tested negative and 7 tested positive for SARS-CoV-2.

**Table.** Summary of the AGPs, Non-AGPs, and Preprocedural Testing until July 20, 2020.

Characteristic	From May 28 to June 15, 2020	From June 16 to July 20, 2020	Total No.
Patients who received consultation only	187	349	536
Procedures recommended	198	266	464
Patients provided non-AGPs and AGPs (no pretesting^a^)	108	162	270
Patients who were advised pretesting^a^	40	154	194
Patients who got tested and were provided AGPs	26	57	83
Patients who tested positive for SARS-CoV-2 (managed as per CDC guidelines)	5	2	7
Infection rate at AKUH, %	19.2	3.5	6.7

AGP, aerosol-generating procedure; AKUH, Aga Khan University Hospital; CDC, Centers for Disease Control and Prevention; SARS-CoV-2, severe acute respiratory syndrome coronavirus 2.

a Pretesting of sample pooling was advised and tracked.

The rate of infection varied in the early and late halves of June 2020. Between May 28 and June 15, 2020, of the 40 patients advised preprocedure testing, 14 patients opted not to get tested and 26 got tested. Consequently, 21 tested negative and 5 tested positive for SARS-CoV-2.

From June 16 to July 20, 2020, the influx of patients had increased, and a total of 154 patients were recommended dental procedures, including AGPs and non-AGPs. Out of these, a total of 57 patients got tested; 55 were negative and only 2 tested positive. The overall positivity rate of the tests done during this period was 6.7%.

## Discussion

The concept of “pool testing” was introduced by Robert Dorfman in 1943 and has been used since for screening infectious diseases (e.g., influenza virus, hepatitis B virus, hepatitis C virus, and human immunodeficiency virus [HIV]) (Hogan et al. 2020). It is a form of group testing, which nowadays is used as a screening tool to identify low-risk or asymptomatic SARS-CoV-2 patients (Hogan et al. 2020).

The aim of this policy development at the dental clinic of our university hospital was to devise a strategy to ensure maximum care to the patients while conserving personal protective equipment (PPE) and minimizing the infection rate among DHCPs who are directly involved in providing patient care. To the best of our knowledge, this strategy has previously been researched for population screening and epidemiological surveys but not as a preprocedural protocol (Hogan et al. 2020). According to our findings, preprocedural testing allowed us to provide safe elective dental treatment to our patients during the pandemic. We were also successful in curtailing transmission of the infection to dentists and auxiliary staff, especially in June 2020, when the national infection rate was at its peak. This had a considerable impact on the prevention of infection in the dental department at Aga Khan University Hospital, where the nosocomial infection rate was maintained to zero (Umer 2020b).

“Pool testing” also addressed patients’ financial concerns, as the pool testing method provides sensitivity similar to the RT-PCR test, with a total cost of $17 at our testing site. The cost of a single pool test is approximately one-third of the total cost for the RT-PCR test at our center. This cost can further decrease by 20-fold with a larger pool size (Mutesa et al. 2020). Furthermore, this strategy conserves reagents and the workforce required for testing without overloading the system in a resource-restrained environment (Wacharapluesadee et al. 2020).

Elective dental treatment was deferred in case the patient tested positive for SARS-CoV-2. These patients are further asked to meet with SARS-CoV-2 monitoring staff, who provided them with basic knowledge, understanding, and particulars of care for the infected individuals. The hospital also had the provision of a negative pressure room equipped with a portable dental unit should there be a need to treat a COVID-19–positive patient for emergency dental work (Umer 2020a).

The patients who opted not to get tested were contacted at a later date over the phone and were asked reasons for not getting tested. The most common reason stated was the fear of getting a positive test result. This behavior is commonly known as willful ignorance or strategic ignorance, in which patients avoid medical diagnosis for the fear of social costs (14-d isolation, stigma, and opportunity cost), which outweigh any benefits of testing. This behavior is also seen in patients with other diseases like HIV or breast cancer (Thunström et al. 2020).

The major limitation of employing pool testing is that it is a type of PCR test with similar disadvantages, such as it is time-consuming and requires a high level of PPE and skilled staff to cater to the amplified risk of infection that comes along with collecting the nasopharyngeal samples (Pan et al. 2020).

Another limitation of our strategy is that the sensitivity and specificity of pool testing were not scrutinized as a head-to-head comparison against gold-standard RT-PCR, as we do not know if sample pooling causes a dilution effect that may negatively affect the diagnostic validity of the test. To investigate this uncertainty, further diagnostic validity studies are required comparing RT-PCR with pool testing. Only 1 study is known to us that conducted a head-to-head comparison and did not find any difference between the pool test and RT-PCR in terms of sensitivity values, but the study was underpowered (Wacharapluesadee et al. 2020).

This protocol also raises a few ethical concerns as a false-negative result may subject the DHCPs to a false sense of security and an increased chance of exposure. In case of a false positive, the patient may require further unneeded testing and/or may need to undergo self-isolation and anxiety.

In our strategy, false negatives were considered true negatives as we did not subject our samples to further testing knowing that a false-negative rate of RT-PCR may range from 2% to 28% (sensitivity of 71%–98%). Therefore, it would be fair to assume that pool testing would also have a similar false-negative rate, if not worse, and we recommend that even with negative pool test results, the DHCPs should follow appropriate PPE protocol (Umer et al. 2020). However, we did have a default mechanism to check for false positives in which a pool with a positive result was rechecked with an individual RT-PCR. Considering that an RT-PCR has high specificity, it would be fair to assume that our positive pool test was a true positive (Watson et al. 2020).

Preprocedural pool testing strategy works best when the disease prevalence is low or moderate and the number of samples in the pool is determined by the disease prevalence; it might not be a useful protocol when disease prevalence is high (Wacharapluesadee et al. 2020). This is because low disease prevalence allows batching a larger pool size. As the prevalence increases, the positivity rate within the pool also increases, and thus the cost-saving benefit of pooling may be negated as the positive pool batches will be required to be retested. Therefore, the CDC recommends that laboratories should monitor disease prevalence according to their positivity rate over the previous 7 to 10 d and accordingly adjust pool sizes (https://www.cdc.gov/coronavirus/2019-ncov/lab/pooling-procedures.html).

According to a study published in the *American Journal of Clinical Pathology*, pool testing can be a useful and cost-saving strategy as long as the incidence of SARS-CoV-2 infection remains below 10%, and in our study, the positivity rate was 6.7% (Abdalhamid et al. 2020).

A further limitation of our study is that we know that COVID-19 infection may cause prolonged RNA shedding even when the patients are not infectious anymore. We did not account for any misclassification of our cases, who could have been postsymptomatic (Xu et al. 2020).

An ethical implication of the preprocedure COVID-19 test is if a patient tests positive, how will the individual privacy be maintained? Of course, the dental team prescribing the test needs to be prompted by a positive test outcome so that the planned procedure can be deferred to a later date. At our institution, by law, we were obliged to share this information with the government, because to mitigate the spread of COVID-19, an early response was critical. Right now, such a response may be justified as we are trying to contain this pandemic. However, in the long run, it can give rise to complex individual privacy issues; hence, the legislature will be required to use aggregate data rather than individual data so that they are not misused. Meanwhile, at a practice level, we recommend that best practice according to local laws be identified and maintained for responsible use of such information. In our institution, for example, personnel assigned to inform and follow up positive patients were public health experts allocated by the local government and were legally bound to government data protection regulations.

Another ethical question that may arise is, if the patient refuses preprocedural testing, can the DHCPs refuse to do AGPs, especially if it is a dental emergency? Is it as simple as saying “no mask, no service”? This concern has not been answered, and when we joined this profession, we accepted a certain degree of risk associated with the trade. Therefore, refusing treatment to patients who opt not to get tested is an idea that may or may not get support from bioethics experts.

## Conclusion

Pool testing within its limitations can be a useful preprocedure test in asymptomatic low-risk patients for AGPs in dentistry, especially when the disease prevalence is low or moderate (<10%). It has the potential of reducing testing costs significantly while conserving reagent and other resources. Preprocedure testing, however, also gives rise to certain ethical concerns that also need to be addressed.

## Author Contributions

F. Umer, contributed to conception, design, data analysis, and interpretation, critically revised the manuscript; A. Arif, contributed to conception, design, data acquisition, and analysis, drafted the manuscript. Both authors gave final approval and agree to be accountable for all aspects of the work.
